# Nipah virus (NiV): a ‘new’ addition to the ever changing landscape of infections

**DOI:** 10.3126/nje.v8i2.23090

**Published:** 2018-06-30

**Authors:** Sruthi James, Indrajit Banerjee, Brijesh Sathian, Edwin van Teijlingen

**Affiliations:** 1 Research Coordinator, Clinical Research, Trauma Surgery, Surgery, Hamad General Hospital, Doha, Qatar.; 2 Associate Professor, Department of Pharmacology, SSR Medical College, Belle Rive, Mauritius; 3 Academic Research Associate, Clinical Research, Trauma Surgery, Surgery, Hamad General Hospital, Doha, Qatar.; 4 Professor, CMMPH, Bournemouth University, Bournemouth, UK.

Public Health professionals and researchers always need to be on their toes as the pool of viruses is forever changing. One recent example of this change is the Nipah virus (NiV). This infection is a type of zoonosis, emerging from the family of Paramyxoviridae, genus Henipavirus (RNA virus) and the natural host is Pteropus fruit bat [1-3]. It is transmitted from infected animals to humans through direct contact and contaminated food like date palm sap. The patients are often asymptomatic and few suffers from acute respiratory infections and fatal encephalitis with a case fatality rate of 45-75% [3].

NiV was identified initially in Malaysia in early 1999 among farmers and people who has close contact with pigs during an outbreak of Encephalitis and respiratory illness. Its name originated from the Malaysian village Sungai Nipah, [1, 2]. During this outbreak, nearly 300 people were believed to be infected with 105 casualties and more than a million pigs were slaughtered to prevent the outbreak from spreading. Although no more cases have been reported in Malaysia [1, 2], it moved to the neighbouring State of Singapore in 1999.

Since its first attacks in Malaysia and Singapore, the NiV has appeared in Siliguri, West Bengal (India) in 2001 with evidence of person-to-person transmission in hospital settings (nosocomial infection) [4]. Sixty six cases with 45 deaths (68% of the cases) were reported in two outbreaks [5]. A further small outbreak of five people occurred in Nadia on 2007 with a 100% fatality rate of five people.

In Bangladesh, several outbreaks of Nipah encephalitis have been reported. A total of 209 cases in 20 districts of whom 161 (77%) died. The first outbreak was in Meherpur (2001) with death rate of nine out of 13 cases. Since then a series of concurrent epidemics occurred untill 2012. In January 2003, eight deaths (n=12) were reported in Naagaon. Two outbreaks in 2004 reported a death rate of 23 (n=31) in Rajbari and 27 (n=36) in Faridpur and 11 out of 12 people lost their lives in the Tangail epidemic of early 2005. From January to April 2007, nine people died three (n=7) from Thakuragaon, five (n=8) from Kushtia, Pabnaand Natore and one (n=3) from Naogaon. Manikgonj sawa 100% (n=5) fatality rate in Feb 2008. In April 2008, Rajbari and Faridpur again suffered from NiV with a mortality rate of five out of seven cases, but in Gaibandha, Rangpur and Nilphamari, no deaths (0%) were reported among three cases. [1, 2].

Without any reduction in the series of epidemic, surprisingly the virus moved to other new places in Bangladesh such as Gopalganj, Madaripur along with Faridpur and Rajbari in 2010 and claiming the lives of 14 of the 16 infected people. Whilst 40 out of 44 registered cases died in Lalmohirhat, Dinajpur, Comilla, Nilphamari and Rangpur in early 2011. The final outbreak was in 2012 in Joypurhat, Rajshshi, Natore, and Rajbari with a fatality rate of 83% (n=12). In all instances, the identified risk factors were the consumption of raw date palm sap and direct contact with infected individuals. Infected fruit-eating bats (Pteropus bats) were identified as a natural host for NiV [6].

In 2015, the Philippines National Epidemiology Centre received a report of 17 persons (11 with encephalitis, 5 with influenza like illness, and 1 with meningitis) and nonfatal infections with concurrent neurologic diseases and abrupt deaths in several horses. The mode of transmission was from direct exposure to infected horses, contact with contaminated body fluids during slaughtering of sick horses, and / or consumption of undercooked meat from infected horses [7]. Recently, in the third NiV outbreak in India, in Kerala 17 people from the districts of Malapuram and Kozhikode died in June 2018 and two recovered completely. Again, it appeared that fruit eating bats were the primary source of infection and the public is warned not to eat fruits already partly eaten by bats.

Since there is currently no effective specific treatment for Nipah virus infection, nor do we have any vaccines against it in humans or in animals, treatment is focused on dealing with the symptoms only.

Recently Favipiravir, a purine analogue antiviral drug which was approved in Japan against influenza strains was found to be effective against the Nipah virus in inhibiting the replication and transcription at micromolar concentration in Syrian hamster model. But Favipiravir needs further evaluation before it can be used for the Nipah viruses [8].

Antifusion lipopeptides has been found to be effective in for prophylaxis of lethal NiV in hamster and non-human primates. Antifusion lipopeptide inhibitors with a desired pharmacokinetic property could be targeted against NiV in future [9].

For Public Health surveillance and multidisciplinary approaches on investigations and therapy and development of vaccines are vital. As with all emerging viruses harmful to humans, an effective system for the detection of infectious diseases as well as for the identification for new causes, risk factors and characteristics in challenged settings is crucial to reduce the disease burden in the population.
